# Prior acute exercise restores postprandial fat oxidation in active cannabis users

**DOI:** 10.14814/phy2.15968

**Published:** 2024-03-07

**Authors:** Matthew M. Schubert, Samantha Terauds, Maren Plant, Grace Handler, Colin Atkins, Casandra Mendez

**Affiliations:** ^1^ Metabolism and Applied Physiology Laboratory, Department of Kinesiology California State University San Marcos California USA; ^2^ School of Medicine George Washington University Washington DC USA

**Keywords:** cannabis, exercise, lipid, metabolism, substrates

## Abstract

Data suggest cannabis users have similar or lower levels of blood lipids compared to nonusers. However, the extent to which cannabis users experience postprandial lipemia is not known. Eleven cannabis users and 11 nonusers completed either rest or 1 h of exercise at their ventilatory threshold the evening before a meal tolerance test (MTT). Substrate oxidation, blood pressure, and capillary blood were obtained before and every 30–60 min post‐meal for 3 h. Linear mixed models were utilized to examine differences in variables between groups, conditions, across time, and their interactions. Exercise led to increased fat oxidation post‐MTT (*p* < 0.05), with cannabis users exhibiting higher AUC compared to the control trial (*p* < 0.05). Exercise also caused significantly lower levels of triglycerides (*p* < 0.05). Metabolic flexibility was improved in cannabis users in the exercise trial only (*p* < 0.05). No effect of group, trial, or interactions were detected for other variables of interest (all *p* > 0.05). This study indicated that prior exercise improves lipid metabolism in cannabis users and nonusers after a high‐fat meal test. Cannabis users appear sensitive to the effects of exercise. Future studies should incorporate additional meals and variables related to cardiovascular health and metabolism.

## Graphical abstract

Cannabis users (CU) and non‐users (NU) underwent two, two‐day trials in a random order consisting of exercise or rest 12–16 h before a meal tolerance test. CU exhibited suppressed fat oxidation post‐meal in the control condition compared to NU, but exercise restored fat oxidation in CU. Triglyceride and cholesterol responses were similar between CU and NU. We conclude that prior exercise restores postprandial fat oxidation in CU.

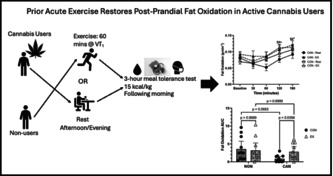

## 1 INTRODUCTION

Cannabis use has a long, ubiquitous history of human consumption. Within the last ~15 years, increased legalization of cannabis for recreational and medicinal purposes has led to explosive growth in legal cannabis markets. Specifically, it was estimated that legal cannabis markets were worth ~$8 billion USD in 2017, with some predicting sales in excess of $24 billion USD by 2025 (Page et al., 2020). The increasingly widespread access to cannabis products raises concerns about the long‐term health consequences of cannabis consumption.

Cardiovascular disease (CVD) remains one of the leading causes of death in industrialized nations. There are many risk factors for CVD, most of which are modifiable. For example, cigarette smoking is a well‐established risk factor due to a variety of substances found in cigarettes. Given the similar chemical composition of cigarette and cannabis smoke (Graves et al., 2020), and the growth of legal cannabis markets, it would seem prudent to examine the cardiovascular and metabolic health of cannabis users (CU). Data are inconsistent, with cross‐sectional studies suggesting some acute adverse health effects and epidemiological studies generally reporting minor or no long‐term adverse effects when comparing CU to non‐users (NU). Many laboratory studies are from the 1960s–70s utilizing smoked cannabis with concentrations of Δ‐9‐tetrahydrocannabinol (THC) that are not reflective of current legal market cannabis (5%–10% THC vs. 15%–90% THC), though these studies have illustrated the potency of THC on sympathetic nervous system activation, which manifests with increased respiratory and heart rates among other responses (Benowitz & Jones, 1975; Weiss et al., 1972). Much of the current literature is observational from longitudinal cohort studies. For example, data from the CARDIA study has reported that cannabis smokers had no increased rates of CVD or CVD risk markers, had no difference in arterial calcium levels, had lower levels of visceral adipose tissue, lower fasting glucose and insulin, and lower waist circumference and body mass index (Auer et al., 2018; Bancks et al., 2018; Jakob et al., 2021; Penner et al., 2013; Reis et al., 2017). Conversely, data from the same study indicated an increased risk of prediabetes (but not diabetes) with current cannabis use or high lifetime exposures (Bancks et al., 2015). It has variously been reported that cannabis users have better HDL cholesterol, lower LDL cholesterol, and lower fasting triglycerides and glucose (Meier et al., 2019; Muniyappa et al., 2013; Ponce Orellana et al., 2015) compared to nonusers. A pair of older studies has reported impaired glucose tolerance with acute intravenous injection and inhalation of cannabis compared to a control condition, but these studies did not compare responses between CU and NU (Hollister & Reaven, 1974; Podolsky et al., 1971). Acute cross‐sectional studies that examined cardiac morphology reported regular cannabis users had increased left ventricular mass, increased stroke volume, increased end‐systolic and end‐diastolic volumes, increased aortic stiffness, and decreased apical rotation (Cheung et al., 2021, 2022). While this might suggest increased risk of developing CVD with cannabis use, data remain limited. Furthermore, it should be noted that most cross‐sectional and epidemiological studies utilized fasting metabolic data, and it has been argued that postprandial assessments may improve risk factor screening (Kolovou et al., 2011; Blaak et al., 2012; Yu et al., 2021).

Postprandial lipemia (PPL) is a common occurrence in Western populations due to the consumption of foods high in sugar and fat; and our meal patterns, which cause us to spend most of our time in a postprandial state (Hurren et al., 2011; Pearson et al., 2022; Ryan et al., 2013). It is characterized by elevated levels of lipids, lipoproteins, inflammatory markers, and insulin after a meal (Hurren et al., 2011; Ryan et al., 2013). Chronic elevations in these biomarkers increases the risk of cardiovascular and metabolic diseases (Pearson et al., 2022). A related issue is metabolic flexibility, which is the body's ability to rapidly switch between substrates (i.e., carbohydrates and fats) to create energy for life (Goodpaster & Sparks, 2017). Individuals who are metabolically flexible can quickly and easily switch between substrates depending on the situation, such as exercise, consumption of a high‐fat meal, or periods of prolonged fasting (Goodpaster & Sparks, 2017). Individuals who are metabolically inflexible seem unable to switch between substrates and end up primarily burning carbohydrate while dietary fat is stored as excess fat mass (Goodpaster & Sparks, 2017; Thompson et al., 2012). A recent study reported an exaggerated PPL response to a high‐fat meal in cigarette smokers compared to nonsmokers (Alotaibi et al., 2021). It is not known how cannabis smoking influences metabolic flexibility or PPL, but one can speculate that similar adverse effects would occur over time given the similar chemical composition of cigarette and cannabis smoke (Graves et al., 2020).

Exercise is a potent treatment for preventing or treating cardiovascular and metabolic diseases. Exercise improves metabolic flexibility and reduces PPL (Pearson et al., 2020, 2022; Rogers et al., 2023). Research in young and active cigarette smokers demonstrated that, when exercise was performed the evening before a meal challenge, the PPL response was attenuated compared to the non‐exercise condition (Alotaibi et al., 2021). Given that cannabis users participate in physical activity at rates similar to other populations (Ong et al., 2021; Smith et al., 2021; YorkWilliams et al., 2019), it seems prudent to examine the metabolic effects of exercise in this population. Thus, the purpose of this study was to determine if cannabis users had altered metabolic flexibility and PPL compared to nonusers, and if cannabis users are sensitive to the effects of exercise on these variables. We hypothesized that cannabis users would have an exaggerated PPL response characterized by elevated triglycerides, and that exercise would not attenuate this response, when compared to nonusers.

## 2 METHODS

**FIGURE 1:**
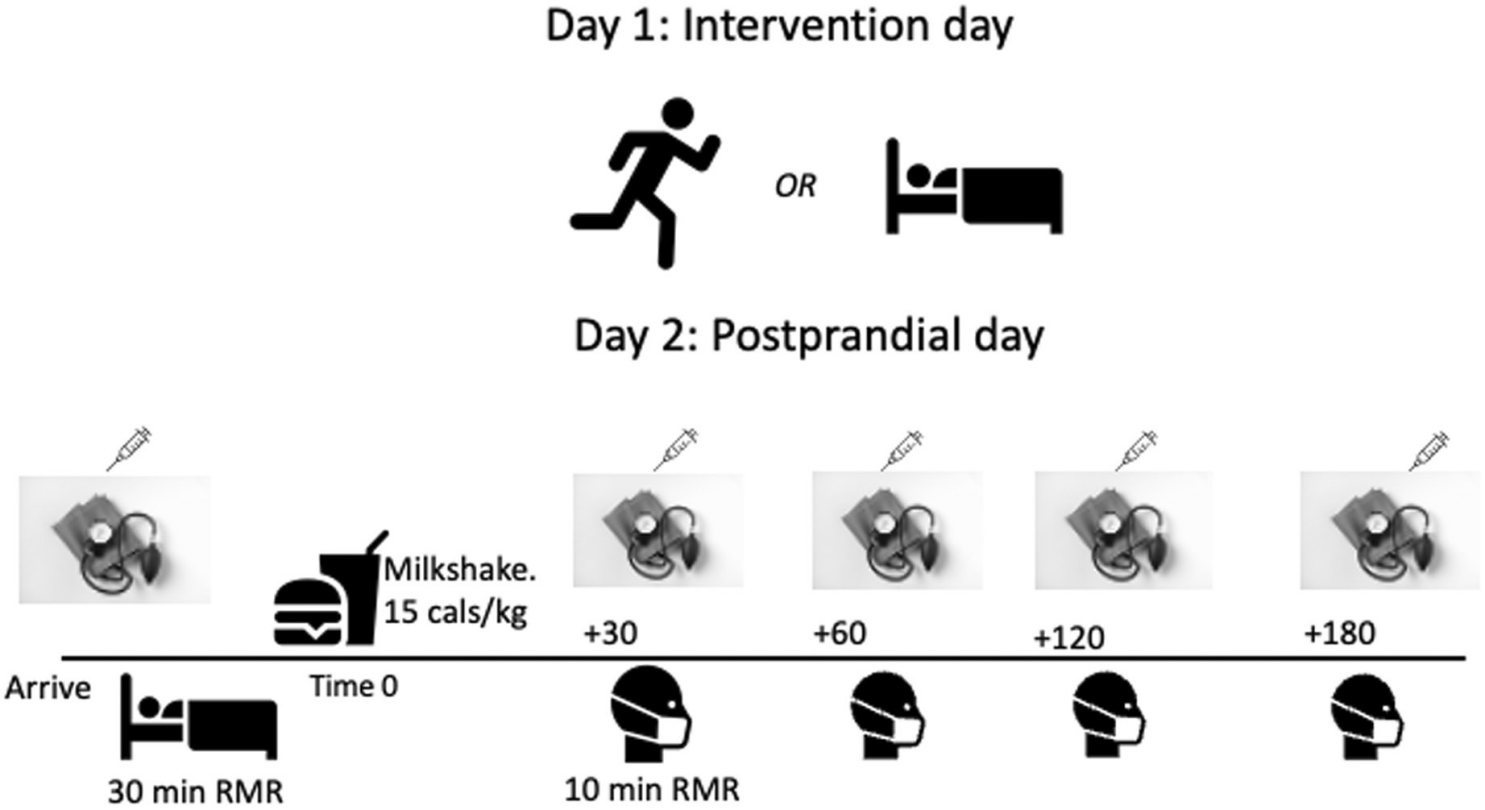
Schematic overview of study. Needles represent capillary blood samples; blood pressure cuff represents mean arterial pressure; and head with mask represents indirect calorimetry.

This study utilized two, 2‐day experimental trials in a random order. Day 1 consisted of rest or 60 min of exercise in the afternoon/evening, while Day 2 (the following morning) incorporated a mixed meal tolerance test (MTT) to evaluate the influence of prior exercise on postprandial lipemia, glucose, substrate oxidation, and blood pressure in cannabis users compared to nonusers. A schematic overview of the study protocol is displayed in Figure 1 below. A total of 30 participants expressed interest in this study. Two participants stopped responding after initial contact, three withdrew after completing the initial survey and before scheduling baseline testing, and three participants withdrew after completing baseline testing. Twenty‐two participants completed the study (*n* = 11 cannabis users and *n* = 11 nonusers) and are included in the analysis. This study was reviewed and approved by the California State University San Marcos Human Subjects Institutional Review Board (#1888533–1). Participants were included if they were physically active (≥ 150 min MVPA per week from self‐report); 18–50 years of age; had no history of cardiovascular or metabolic disease; and were not taking medications impacting metabolism aside from birth control. Cannabis users were classified as regular users if they used ≥1×/week for at least 1 year. Participants were compensated $200 USD at the conclusion of the study. Participant characteristics are displayed in Table 1.

**TABLE 1:** Participant characteristics by cannabis use status.

Variables	Nonusers (NU, *n* = 11, five males)	Cannabis users (CU, *n* = 11, six males)	*p*‐Value
Age (years)	25.4 ± 5.1	28.9 ± 7.2	0.197
Height (cm)	170 ± 10	170 ± 10	0.910
Mass (kg)	67.2 ± 11.9	69.3 ± 15.5	0.733
BMI	23.1 ± 2.4	23.6 ± 3.1	0.717
Body fat (%)	19.7 ± 7.6	19.6 ± 8.0	0.978
Fat mass (kg)	13.1 ± 5.7	13.8 ± 7.1	0.795
Lean mass (kg)	54.1 ± 12.6	55.5 ± 12.3	0.807
VO_2_peak (mL⋅kg^−1^⋅min^−1^)	45.2 ± 7.6	43.7 ± 10.1	0.710
VO_2_ @ VT1 (mL⋅kg^−1^⋅min^−1^)	31.0 ± 6.2	32.3 ± 6.3	0.641
Percent VO_2_peak @ VT	69.3 ± 10.9	74.9 ± 10.2	0.227

*Note*: Group differences were analyzed with independent *t*‐tests. Between‐group differences were examined using independent samples t‐tests. Data are means ± SD.

Abbreviations: BMI, body mass index; CU, cannabis users; NU, nonusers; VT, ventilatory threshold.

### 2.1 Power calculation

Based on data from prior research, we estimated that to detect a within‐group moderate effect (Cohen's *d* = 0.40) of prior exercise on postprandial TG AUC, a minimum of 20–24 participants (*n* = 10–12 per group) would be necessary. Moderate‐to‐large within‐group effects were detected with 22 participants (*n* = 11 per group).

### 2.2 Initial testing

After providing written informed consent, participants completed an electronic questionnaire for health history, physical activity habits, and tobacco, cannabis, and alcohol use to determine study eligibility. Once eligibility was confirmed, participants attended the laboratory for a baseline visit. Participants were asked to limit physical activity before their visit, to drink 500–600 mL of water in the 2–3 h beforehand, and not to consume food, tobacco, cannabis, or caffeine less than 3 h before their visit. This visit consisted of assessments of height and weight, body composition, and maximal aerobic fitness. Height was measured via stadiometer and body mass and composition were assessed with multifrequency bioelectrical impedance (InBody 770, InBody USA, Cerritos, CA, USA). Participants then placed a heart rate monitor on their chest (Garmin HRM, Garmin Inc., Olathe, KS, USA) and completed a 5‐min self‐paced warm‐up on a motorized treadmill (4Front, Woodway Inc., Waukesha, WI, USA). After warming up, VO_2_peak was determined via an incremental treadmill test to volitional exhaustion. Breath‐by‐breath data were filtered with values >4 standard deviations from the surrounding values excluded and presented as 10‐breath rolling averages (Cosmed Quark RMR/CPET, Cosmed Inc., Italy). Participants ran or walked against a constant speed while the incline increased 1% every 1 min to exhaustion. The oxygen consumption at the ventilatory threshold (VT) was determined using the V‐slope method (Beaver et al., 1986). VO_2_peak was calculated as the average of the final 30 s of exercise. VO_2_peak was confirmed with at least two of the following criteria: rate of perceived exertion (RPE) ≥17; heart rate ± 10 of age‐predicted maximum; respiratory exchange ratio (RER) ≥ 1.10; and/or a plateau in VO_2_ < 2 mL⋅kg^−1^⋅min^−1^ (Robergs et al., 2010).

After baseline testing, participants were randomly assigned to complete either a control trial or an exercise trial, followed by the opposite condition 4–7 days later where feasible. Randomization sequences were generated using a website (Randomizer.org). Where possible, female participants completed testing during the first 2 weeks of their menstrual cycle or oral contraceptive pill regimen (nonusers *n* = 4; cannabis *n* = 3). If this was not possible, visits were scheduled approximately 1 month aside to account for variations in metabolism across menstrual cycle phases (nonusers *n* = 2; cannabis *n* = 2).

### 2.3 Control and standardization

For the control trial, the participants did not attend the laboratory the day before the MTT and reported to the laboratory in the morning (0530–0930) after an overnight fast and abstention from exercise. All participants refrained from cannabis, tobacco, alcohol, and caffeine for at least 12 h before each MTT. Dietary intake was recorded via food diaries prior to the first trial, and this was replicated for the second trial. Intake data were analyzed using the National Cancer Institute's Automated Self‐Administered 24‐hour dietary assessment tool (ASA‐24). Participants were asked to limit their activity 24 h before each MTT (except for the exercise trial), including avoiding stairs, structured and unstructured exercise, and ambulating as little as possible both the evening before and morning of each MTT. Compliance was confirmed with verbal questioning prior to each MTT. Energy and macronutrient intakes of the participants before each trial were well matched (One‐way repeated‐measures ANOVA *p* = 0.517. Cannabis users: Con = 2874 ± 431 kcal; Ex = 2795 ± 502 kcal, *p* = 0.726. Nonusers: Control = 2754 ± 467; Ex: 2835 ± 507 kcal, *p* = 0.437).

### 2.4 Exercise trial

In the exercise trials, participants reported to the laboratory 12–16 h before their MTT (1500–1800) and completed 60 min of moderate‐to‐vigorous intensity exercise at ~100% of their VT. Gas exchange data were collected for the first 15 min of exercise, 25–35 min, and 50–60 min while heart rate (Garmin HRM, Garmin Inc., Olathe, KS, USA) was monitored continuously (Cosmed Quark RMR/CPET, Cosmed Inc., Italy). Intensity (speed/incline) was adjusted as necessary to keep VO_2_ between 95% and 105% of VT. Before exercise and every 5 min during exercise, RPE (Borg, 1982) and positive/negative affect (Hardy & Rejeski, 1989) were measured using validated scales. Immediately before and after exercise, a capillary blood sample was also collected from the index finger for analysis of blood lactate (Lactate Plus, #40813, Nova Biomedical, Waltham, MA, USA). When the face mask was removed during exercise, participants were given the option to drink 150 mL of water. Finally, 10 min post‐exercise, participants completed an exercise enjoyment questionnaire (Kendzierski & DeCarlo, 1991). Gas exchange data were averaged over the final 5 min of each collection period, with rates of carbohydrate and fat oxidation calculated using published stoichiometric equations for exercise (Jeukendrup & Wallis, 2005).

### 2.5 Experimental trials

Upon arrival for the MTT, participants were weighed (Seca Model 874). A sample of capillary blood (50 μL) was collected into a heparinized capillary tube and immediately (within 5 min) analyzed for triglycerides, glucose, total cholesterol, LDL cholesterol, and HDL cholesterol using a Cholestech LDX analyzer (Lipid Profile‐Glucose test cassettes, #97991; Abbott, Carlsbad, CA, USA). The analyzer was calibrated with each new lot of test cassettes using calibration solutions and test cassettes purchased from the manufacturer; the optics were calibrated each day before use using the provided cassette. Participants then rested supine while a blood pressure cuff was placed on the left arm and a facemask (Hans Rudolph V2) connected to a metabolic cart via flow turbine and sampling line was attached to their face. Twenty minutes of gas exchange data were collected to estimate resting metabolic rate (Cosmed Quark RMR/CPET, Cosmed Inc., Italy). Blood pressure was measured (Tango M2, Suntech Inc., Cary, NC, USA) in duplicate after 10 min of rest with 4 min between measurements and mean arterial pressure was calculated as diastolic blood pressure added to one third of the difference between systolic blood pressure and diastolic blood pressure. The final 5–10 min of the gas exchange data were used to calculate energy expenditure and substrate oxidation after ensuring a steady state (< 10% variation in RER, VO_2_, and VCO_2_). Rates of carbohydrate and fat oxidation were calculated using published stoichiometric equations for resting conditions (Frayn, 1983). Thirty, 60, 120, and 180 min after the MTT, capillary blood, blood pressure, and gas exchange data were collected using identical procedures. Participants were free to consume water ad libitum during trials.

### 2.6 Meal energy and nutrient contents

Each participant's mass was entered into a spreadsheet which provided information on the energy content of the participants' MTT along with the calculated weights of various ingredients to be mixed to achieve the desired energy content. The MTT was a milkshake consisting of vanilla or chocolate ice cream (Häagen Dazs, Minneapolis, MN, USA), whole milk (Kroger, Cincinnati, OH, USA), and heavy whipping cream (Kroger, Cincinnati, OH, USA). All shakes were prepared using the same 1000‐watt blender (Ninja BL610, SharkNinja, Inc., Needham, MA, USA) and standardized for blend speed and time. The MTT delivered 15 kcal·kg^−1^ body mass. The milkshake contained approximately 55% energy from fat (~62.5 g), 30% energy from carbohydrate (~76.7 g), and 15% protein (~38.3 g). The shake contained 21.5 g of added sugars (~8.4% energy). The control trial MTT energy was 1025 ± 205 kcal versus 1020 ± 204 kcal for the exercise trial (*p* = 0.227). Energy from fat (*p* = 0.180), carbohydrates (*p* = 0.168), protein (*p* = 0.365), and added sugars (*p* = 0.213) were not different between trials. The energy content of the meal was equivalent to 35% of the participants' habitual daily energy intake.

### 2.7 Statistical analysis

Data comparing differences between participant characteristics and exercise data were analyzed using independent *t*‐tests. For triglycerides, glucose, total cholesterol, LDL cholesterol, HDL cholesterol, RER, fat oxidation, and mean arterial pressure, incremental area‐under‐the‐curve data were calculating using a published spreadsheet. The iAUC was chosen as the summary statistic of interest as this expresses the total relative change from baseline during the postprandial period (Narang et al., 2020). Metabolic flexibility was calculated for each trial, based on prior research, as the fasting RER subtracted from the average postprandial RER (Gilbertson et al., 2018). Data were analyzed using Prism 9.0 (GraphPad Software, La Jolla, CA, USA) with linear mixed models with trial (control vs. exercise), group (nonuser vs. user), and time (Baseline, 30, 60, 120, and 180 min) as fixed factors. The Greenhouse–Geisser correction was applied when sphericity was violated. Post hoc comparisons were conducted with Bonferroni's multiple comparison tests. For some comparisons, pairwise effect sizes (control vs. exercise; nonuser vs. user) were calculated as Cohen's *d*, and these are reported as small (0.2), moderate (0.5), and large (0.8) effects, respectively. Data in tables are presented as mean ± standard deviation (SD) and data presented in figures are mean ± standard error of the measurement (SEM) to avoid distortion on line graphs and mean ± 95% confidence intervals on bar graphs. Statistical significance was accepted when *p* < 0.05.

## 3 RESULTS

### 3.1 Participants

Participant characteristics are displayed in Table 1. There were no differences for age, mass, body composition, oxygen uptake, or ventilatory threshold data.

### 3.2 Cannabis use

Cannabis users had a median age of first use of 18 years (range = 13–20) and median age of regular use of 19 years (range = 16–33). Thus, cannabis users had approximately 10 years of use history. In the prior 30 days, cannabis use ranged from 6 to 30 days, with a median of 25 use‐days. Median use per day was 2.5 uses with a range of 1–6 uses. Participants primarily smoked flower (*n* = 6), used oral formulations (*n* = 3), or vapes and dabs (*n* = 2). One cannabis user reported packing their cannabis “bowls” with 25%–30% tobacco; no other cannabis users reported tobacco consumption.

### 3.3 Exercise trials

Data from the exercise bout for both participant groups are in Table 2. VO_2_ was matched between trials, although the control group exercised at a lower percentage of their VT (*p* = 0.04, *d* = 0.93). Heart rate, RER, substrate oxidation, total energy expenditure, RPE, enjoyment (PACES), and lactate were not different between the groups. Observationally, most cannabis users (*n* = 7) walked up an incline while most nonusers (*n* = 8) jogged without the need for manipulating the incline, though the between‐group differences for speed and grade were not significantly different.

**TABLE 2:** Exercise responses.

Variables	NU (*n* = 11, five males)	CU (*n* = 11, six males)	*p*‐value
Exercise VO_2_ (mL⋅kg^−1^⋅min^−1^)	29.4 ± 6.0	32.4 ± 5.7	0.236
Percent VT	95.2 ± 4.9	100.5 ± 6.4	0.040
Exercise RER	0.91 ± 0.03	0.92 ± 0.03	0.874
Exercise Carbohydrate oxidation (g)	116.8 ± 49.1	127.8 ± 37.7	0.561
Exercise fat oxidation (g)	16.9 ± 6.1	19.7 ± 9.0	0.405
Total energy expenditure (kcal)	618 ± 198	689 ± 187	0.399
Exercise heart rate (bpm)	167 ± 10	162 ± 10	0.267
Rate of perceived exertion (6–20)	12.9 ± 1.2	12.5 ± 1.3	0.506
Pre‐exercise blood lactate (mmol⋅L^−1^)	1.38 ± 0.45	1.12 ± 0.28	0.112
Post‐exercise blood lactate (mmol⋅L^−1^)	3.54 ± 1.21	3.11 ± 0.84	0.348
Treadmill speed (km⋅h^−1^)	9.01 ± 0.93	7.88 ± 1.90	0.24
Treadmill grade (%)	0.25 ± 0.40	1.40 ± 1.80	0.069
Enjoyment (0–126)	96 ± 5	98 ± 4	0.743

*Note*: Data are means ± SD. Group differences were analyzed with independent *t*‐tests. Between‐group differences were examined using independent samples *t*‐tests.

Abbreviations: CU, cannabis users; NU, nonusers.

### 3.4 Fasting data

Fasting data for primary variables prior to each MTT are shown in Table 3. There were no significant differences between trials or groups for any variable at the baseline time point for all MTTs.

**TABLE 3:** Fasting data prior to each MTT.

Variables	NU (*n* = 11, five males)		CU (*n* = 11, six males)		*p*‐Value
	CON	EX	CON	EX	
Fasting RER	0.83 ± 0.04	0.80 ± 0.03	0.82 ± 0.05	0.81 ± 0.05	0.439
Fasting carbohydrate oxidation (g⋅min^−1^)	0.16 ± 0.06	0.13 ± 0.04	0.14 ± 0.06	0.12 ± 0.06	0.784
Fasting fat oxidation (g⋅min^−1^)	0.07 ± 0.02	0.09 ± 0.03	0.08 ± 0.03	0.09 ± 0.03	0.454
Fasting energy expenditure (kcal⋅min^−1^)	1.25 ± 0.31	1.32 ± 0.28	1.27 ± 0.31	1.31 ± 0.33	0.721
Fasting triglycerides (mmol⋅L^−1^)	0.95 ± 0.19	0.90 ± 0.15	0.77 ± 0.18	0.61 ± 0.13	0.093
Fasting glucose (mmol⋅L^−1^)	4.81 ± 0.58	4.74 ± 0.74	4.65 ± 0.33	4.47 ± 0.44	0.728
Fasting total cholesterol (mmol⋅L^−1^)	3.86 ± 1.00	3.79 ± 0.99	4.58 ± 1.29	4.56 ± 1.30	0.994
Fasting LDL cholesterol (mmol⋅L^−1^)	2.30 ± 0.88	2.08 ± 0.87	2.77 ± 0.95	2.73 ± 1.01	0.779
Fasting HDL cholesterol (mmol⋅L^−1^)	1.35 ± 0.31	1.41 ± 0.29	1.38 ± 0.36	1.44 ± 0.47	0.992
Fasting SBP (mm Hg)	119 ± 8	119 ± 8	120 ± 11	120 ± 10	0.907
Fasting DBP (mm Hg)	71 ± 6	74 ± 5	69 ± 6	68 ± 6	0.349
Fasting MAP (mm Hg)	87 ± 6	89 ± 5	86 ± 6	85 ± 6	0.472
Fasting heart rate (bpm)	54 ± 5	53 ± 8	54 ± 7	56 ± 6	0.563

*Note*: Data are means ± SD. Data were analyzed with a one‐way repeated measures ANOVA. Differences between groups and trials were examined using a one‐way repeated‐measures ANOVA.

Abbreviations: CU, cannabis users; NU, nonusers.

### 3.5 Primary variables

**FIGURE 2:**
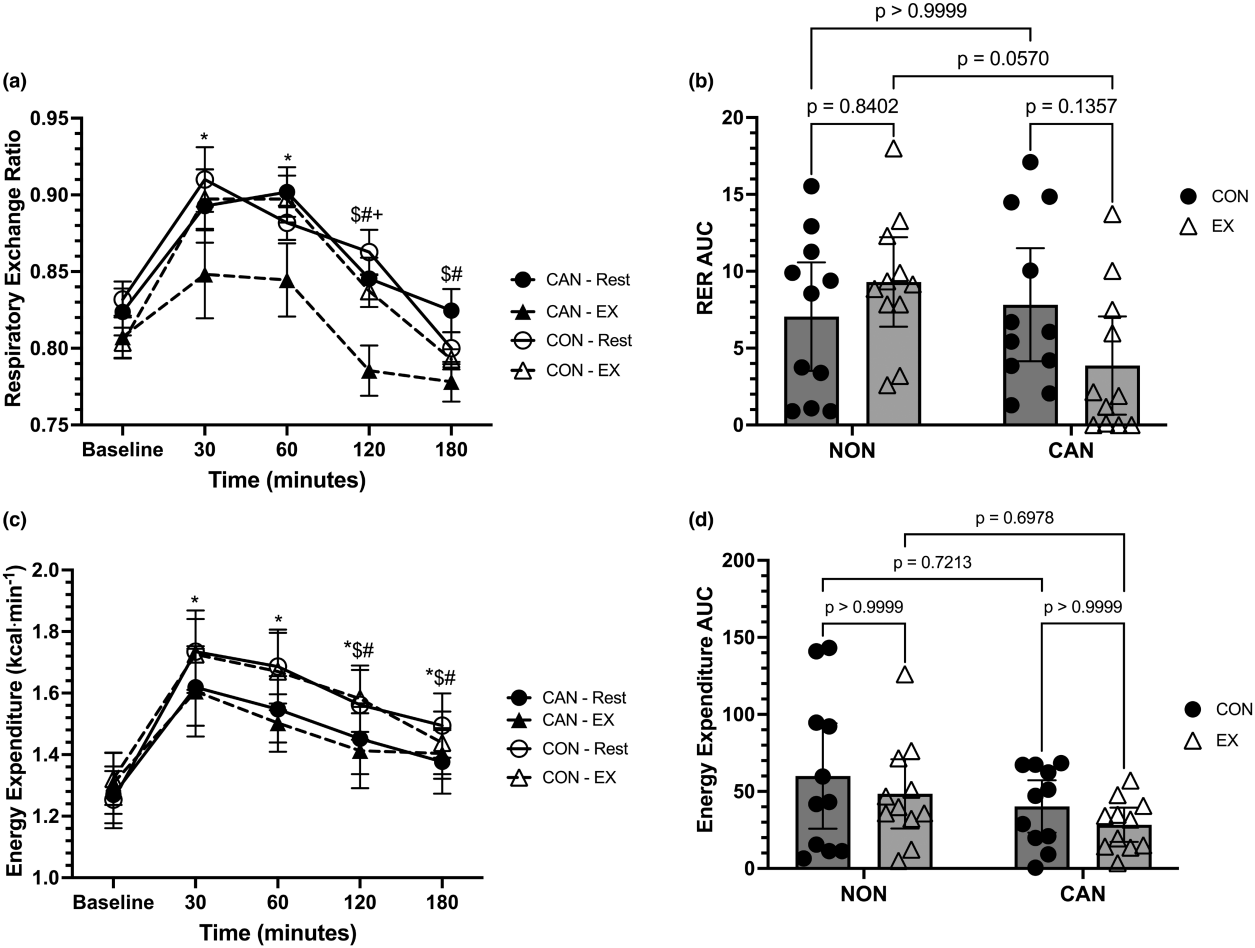
(a) Respiratory exchange ratio (RER) responses over time. Data were analyzed with a linear mixed model. Time effects denoted by * (different from Baseline, *p* < 0.05), $ (different from 30 min, *p* < 0.05), and # (different from 60 min, *p* < 0.05). Trial effects denoted by + (different from 180 min in Rest only). Data are means ± SEM. (b) RER incremental area under the curve (iAUC). Data were analyzed with a two‐way repeated measures ANOVA. Data are means ±95% confidence intervals. (c) Energy expenditure (EE) responses over time. Data were analyzed with a linear mixed model. Time effects denoted by * (different from Baseline, *p* < 0.05), $ (different from 30 min, *p* < 0.05), and # (different from 60 min, *p* < 0.05). Data are means ± SEM. (d) EE iAUC. Data were analyzed with a two‐way repeated measures ANOVA. Data are means ±95% confidence intervals.

**FIGURE 3:**
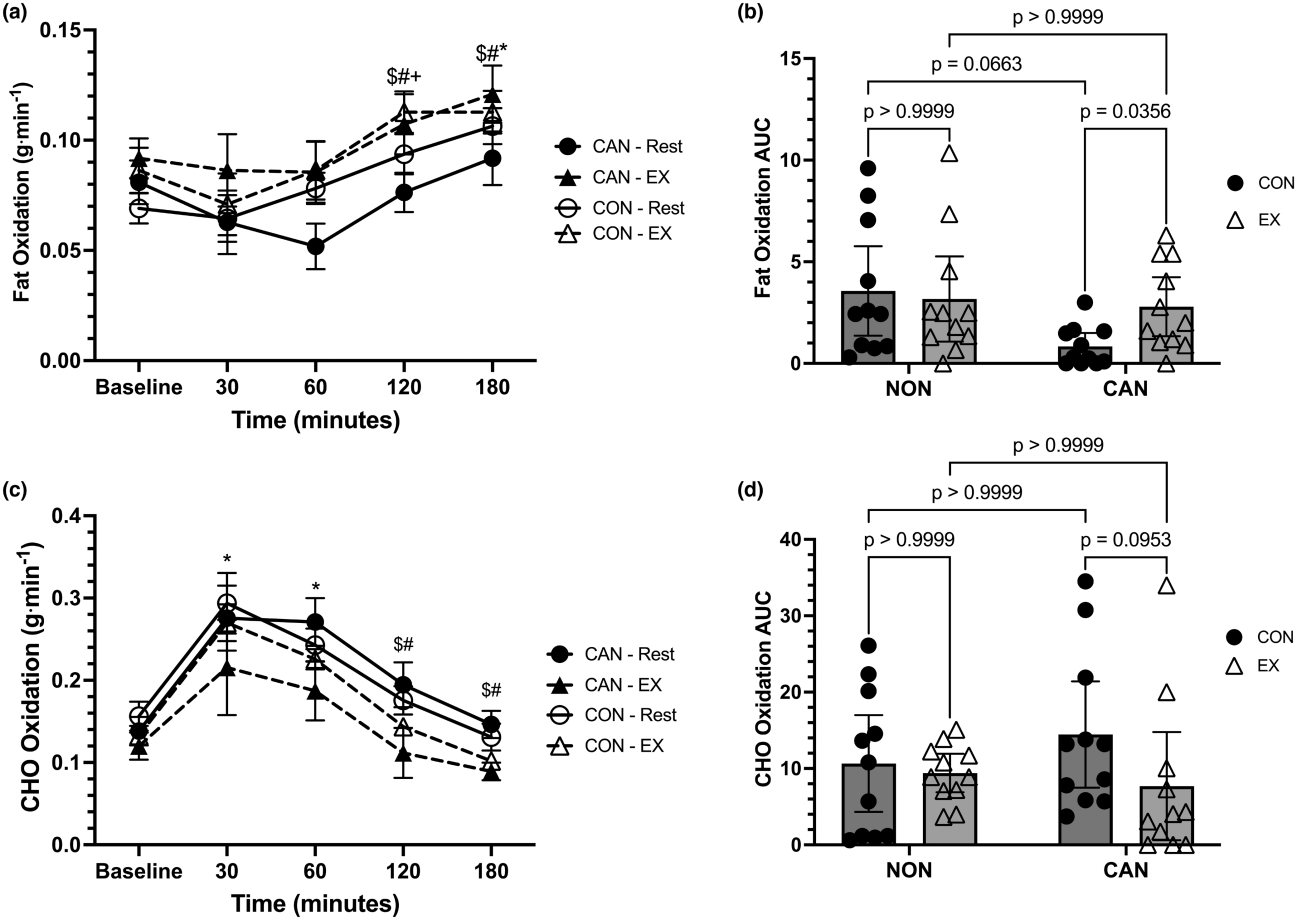
(a) Fat oxidation (FOX) responses over time. Data were analyzed with a linear mixed model. Time effects denoted by * (different from Baseline, *p* < 0.05), $ (different from 30 min, *p* < 0.05), and # (different from 60 min, *p* < 0.05). Trial effects denoted by + (different from Baseline in Exercise only). Data are means ± SEM. (b) FOX iAUC. Data were analyzed with a two‐way repeated measures ANOVA. Data are means ±95% confidence intervals. (c) Carbohydrate oxidation (CHO) responses over time. Data were analyzed with a linear mixed model. Time effects denoted by * (different from Baseline, *p* < 0.05), $ (different from 30 min, *p* < 0.05), # (different from 60 min, *p* < 0.05). Data are means ± SEM. (d) CHO iAUC. Data were analyzed with a two‐way repeated measures ANOVA. Data are means ±95% confidence intervals.

**FIGURE 4:**
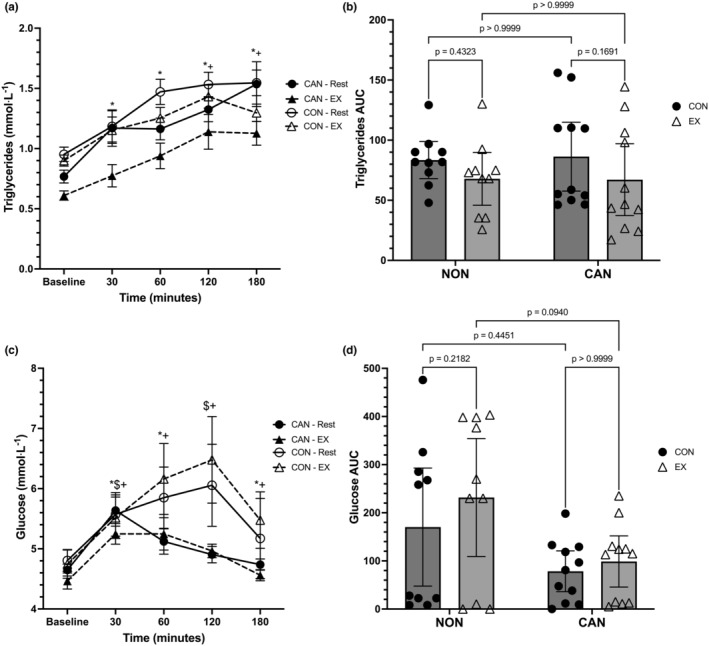
(a) Triglyceride responses over time. Data were analyzed with a linear mixed model. Time effects denoted by * (different from Baseline, *p* < 0.05) and $ (different from 30 min, *p* < 0.05). Trial effects denoted by + (different from 30 min in Exercise only). Data are means ± SEM. (b) Triglyceride iAUC. Data were analyzed with a two‐way repeated measures ANOVA. Data are means ±95% confidence intervals. (c) Glucose responses over time. Data were analyzed with a linear mixed model. Time effects in CU denoted by * (different from Baseline, *p* < 0.05) and $ (different from 180 min, *p* < 0.05). Time effects in NU denoted by + (different from Baseline, *p* < 0.05). Data are means ± SEM. (d) Glucose iAUC. Data were analyzed with a two‐way repeated measures ANOVA. Data are means ±95% confidence intervals.

**FIGURE 5:**
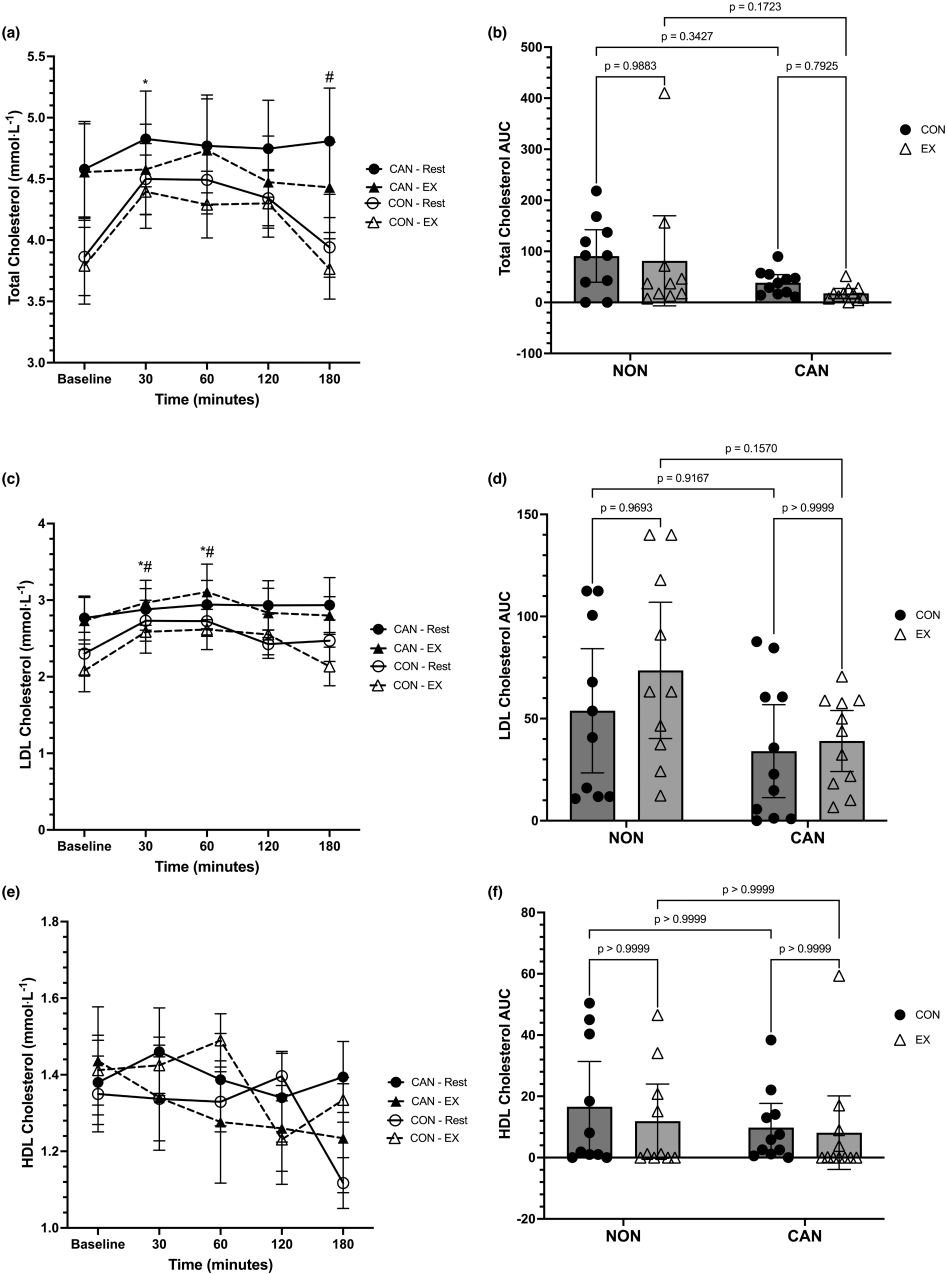
(a) Total cholesterol (TC) responses over time. Data were analyzed with a linear mixed model. Time effects in NU denoted by * (different from Baseline, *p* < 0.05) and # (different from 120 min). Data are means ± SEM. (b) TC iAUC. Data were analyzed with a two‐way repeated measures ANOVA. Data are means ±95% confidence intervals. (c) Low‐density lipoprotein cholesterol (LDL) responses over time. Data were analyzed with a linear mixed model. Time effects denoted by * (different from Baseline, *p* < 0.05). Trial effects denoted by # (different from 180 min, *p* < 0.05). Data are means ± SEM. (d) LDL iAUC. Data were analyzed with a two‐way repeated measures ANOVA. Data are means ±95% confidence intervals. (e) High‐density lipoprotein cholesterol (HDL) responses over time. Data were analyzed with a linear mixed model. (f) HDL iAUC. Data were analyzed with a two‐way repeated measures ANOVA. Data are means ±95% confidence intervals.

**FIGURE 6:**
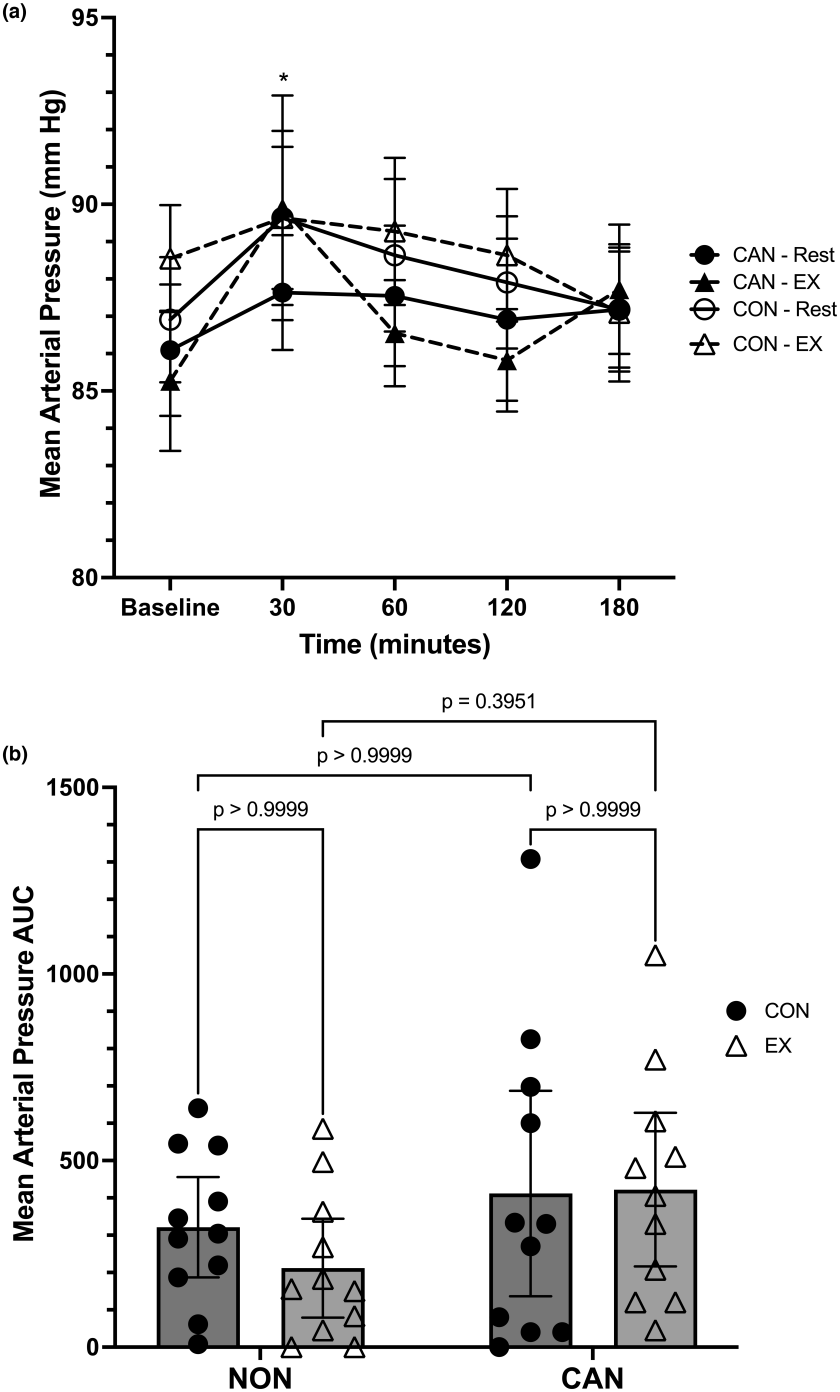
(a) Mean arterial pressure (MAP) responses over time. Data were analyzed with a linear mixed model. Time effects in NU denoted by * (different from 180 min, *p* < 0.05). Data are means ± SEM. (b) MAP iAUC. Data were analyzed with a two‐way repeated measures ANOVA. Data are means ±95% confidence intervals.

Time‐series and iAUC data are displayed in Figures 2, 3, 4, 5, 6. Blood was unable to be obtained from one nonuser participant; thus, for blood sample data, *n* = 10 for non‐users and *n* = 11 for cannabis users.

Indirect calorimetry data are displayed in Figure 2a–d and Figure 3a–d. For RER, a linear mixed model unveiled a main effect of time (*F* = 31.35, *p* < 0.0001), a main effect of trial (*F* = 11.15, *p* = 0.0077), but no main effect of group (*F* = 1.148, *p* = 0.2967). The interactions for time*trial (*F* = 0.7854, *p* = 0.4887), time*group (*F* = 1.613, *p* = 0.1791), trial*group (*F* = 3.861, *p* = 0.0635), and time*trial*group (*F* = 2.363, *p* = 0.06) were not significant (Figure 2a). The trial main effect showed a significantly lower RER in the EX compared to the control trial (mean difference = −0.02827, 95% CI: 0.003–0.054, *p* = 0.0292). In both the exercise and control trials, Baseline RER was significantly less than RER at 30 and 60 min (*p* < 0.05 for all). Further, RER at 30 and 60 min was significantly higher than RER at 120 and 180 min in both trials (*p* < 0.05 for all), with RER higher at 120 min than 180 min in the control trial only (*p* < 0.05). A two‐way repeated‐measures ANOVA for RER iAUC data revealed no effects of group (*F* = 1.822, *p* = 0.1922) or trial (*F* = 0.4821, *p* = 0.4955), though a significant group*trial interaction (*F* = 6.38, *p* = 0.0201) was observed. However, post hoc comparisons were not significant (Figure 2b).

Energy expenditure in kilocalories per minute (EEm) was analyzed using a linear mixed model, which unveiled a main effect for time (*F* = 30.56, *p* < 0.0001). However, the main effects for trial (*F* = 0.0077, *p* = 0.7966), group (*F* = 0.4734, *p* = 0.4993), and the time*trial (*F* = 0.6399, *p* = 0.6093), time*group (*F* = 1.311, *p* = 0.2731), trial*group (*F* = 0.015, *p* = 0.9026), and time*trial*group (*F* = 0.4626, *p* = 0.7629) interactions were not statistically significant (Figure 2c). In both the control and exercise trials, Baseline EEm was significantly less than all other time points (p < 0.025 vs all). Further, in both trials, EEm was higher at 30 and 60 min compared to 120 and 180 min (*p* < 0.035 for all). A two‐way repeated‐measures ANOVA for EEm iAUC data revealed no effect of group (*F* = 0.2.562, *p* = 0.1251), trial (*F* = 2.469, *p* = 0.1318), or group*trial interaction (*F* = 0.00034, *p* = 0.9854) (Figure 2d).

Regarding fat oxidation (FOX), a linear mixed model unveiled a main effect of time (*F* = 13.11, *p* < 0.0001), a main effect of trial (*F* = 14.30, *p* = 0.0097), but no main effect of group (*F* = 0.0423, *p* = 0.8393). The interactions for time*trial (*F* = 0.7812, *p* = 0.5083), time*group (*F* = 1.415, *p* = 0.2367), trial*group (*F* = 2.09, *p* = 0.1637), and time*trial*group (*F* = 1.549, *p* = 0.1959) were not significant (Figure 3a). The main effect for trial was not significant when post hoc analysis was conducted (mean difference = +0.01855, 95% CI: −0.00016 to 0.0373, *p* = 0.0519). In both the exercise and control trials, Baseline FOX was significantly lower than FOX at 180 min, while Baseline FOX was also lower than FOX at 120 min in the exercise trial (*p* < 0.05 for all). In both the exercise and control trials, FOX at 30 and 60 min was significantly lower than FOX at 120 and 180 min (*p* < 0.05 for all). A two‐way repeated‐measures ANOVA for FOX iAUC data revealed no effect of group (*F* = 2.511, *p* = 0.1288) or trial (*F* = 2.665, *p* = 0.1182), but a significant group*trial interaction was observed (*F* = 6.076, *p* = 0.0229). Post hoc comparisons revealed that CU had a lower FOX iAUC in the control compared with the exercise trial (mean difference = −1.946, 95% CI: −3.79 to −0.1027, *p* = 0.0356) (Figure 3b). The comparison for the control condition FOX iAUC between CU and NU did not achieve statistical significance (mean difference = −2.723, 95% CI: −5.57 to 0.1245, *p* = 0.0663).

Carbohydrate oxidation (CHO) was analyzed using a linear mixed model, which unveiled a main effect for time (*F* = 31.17, *p* < 0.0001), a main effect of trial (*F* = 11.87, *p* = 0.0166), but no main effect of group (*F* = 0.2648, *p* = 0.6125). The interactions for time*trial (*F* = 0.8338, *p* = 0.4401), time*group (*F* = 0.42, *p* = 0.7938), trial*group (*F* = 2.2023, *p* = 0.1704), and time*trial*group (*F* = 0.8137, *p* = 0.5201) were not significant (Figure 3c). The main effect of trial revealed that CHO was higher in the control trial compared to the exercise trial (mean difference = +0.043, 95% CI: 0.0045–0.081, *p* = 0.0294). In both the control and exercise trials, Baseline CHO values were lower than 30 and 60 min (*p* < 0.05). In addition, the 30 and 60 min time points in both control and exercise trials were significantly greater than 120 and 180 min (*p* < 0.05 for all). A two‐way repeated‐measures ANOVA for CHO iAUC data revealed no effect of group (*F* = 0.101, *p* = 0.7359), trial (*F* = 4.197, *p* = 0.0538), or group*trial interaction (*F* = 1.989, *p* = 0.1738) (Figure 3d).

Metabolic flexibility (MetFlex) data was analyzed using a two‐way repeated‐measures ANOVA. MetFlex was calculated as Baseline RER minus the average postprandial RER (30, 60, 120, and 180 min). No main effect of group (*F* = 1.865, *p* = 0.1872) or trial (*F* = 0.7287, *p* = 0.4034) were observed; however, a significant trial*group (*F* = 10.29, *p* = 0.0044) interaction was detected. MetFlex values were 0.043 ± 0.035 (CU Control), 0.007 ± 0.04 (CU Exercise), 0.032 ± 0.042 (NU Control), and 0.053 ± 0.027 (NU Exercise). Specifically, CU had lower MetFlex in the exercise trial compared to NU (mean difference: −0.046, 95% CI: −0.086 to −0.005, *p* = 0.0217) and CU had a lower MetFlex in the exercise compared to the control trial (mean difference: −0.036, 95% CI: −0.069 to −0.0016, *p* = 0.0377).

Data from the capillary blood variables are displayed in Figures 4a–d and 5a–f. For triglycerides (TGs), a linear mixed model revealed a main effect of time (*F* = 29.64, *p* < 0.0001), a main effect of trial (*F* = 29.45, *p* = 0.0002), but no main effect of group (*F* = 3.894, *p* = 0.0632). The interactions for time*trial (*F* = 1.885, *p* = 0.1535), time*group (*F* = 0.9041, *p* = 0.4659), trial*group (*F* = 3.745, *p* = 0.068), and time*trial*group (*F* = 1.17, *p* = 0.3310) were not significant (Figure 4a). The main effect of trial revealed that TGs were higher in the control trial compared to the exercise trial (+0.2056, 95% CI: 0.0283–0.383, *p* = 0.0242). In both the control and exercise trials, Baseline TGs were significantly lower than all other time points (*p* < 0.025 for all). In the exercise trial only, TGs were also significantly lower at 30 min compared to 120 (*p* = 0.0032) and 180 min (*p* = 0.0249). A two‐way repeated‐measures ANOVA for TG iAUC data revealed a main effect of trial (*F* = 7.414, *p* = 0.0135), no effect of group (*F* = 0.005, *p* = 0.9438), or group*trial interaction (*F* = 0.0794, *p* = 0.7811). TG iAUC was significantly higher in the control trials compared to the exercise trials (+17.39, 95% CI: 4.024–30.76, *p* = 0.0135) (Figure 4b).

Glucose (GLU) was analyzed using a linear mixed model, which unveiled a main effect for time (*F* = 8.312, *p* < 0.0001), but no main effects for group (*F* = 2.321, *p* = 0.1441) or trial (*F* = 0.247, *p* = 0.5242). The interactions for time*trial (*F* = 1.541, *p* = 0.2225), group*trial (*F* = 4.158, p = 0.0556), and time*trial*group (*F* = 0.192, *p* = 0.9421) did not reach statistical significance; however, there was a significant time*group interaction (*F* = 2.863, *p* = 0.0288) (Figure 4c). The group*trial interaction revealed a lower mean GLU value for CU compared to NU (mean difference: −0.63, 95% CI: −1.22 to −0.043, *p* = 0.036). CU had lower GLU at Baseline compared to 30 and 180 min (*p* < 0.035 for both); furthermore, CU had lower GLU at 30 and 120 min versus 180 min (*p* < 0.03 for both). In NU, Baseline GLU was significantly lower than all other time points except 180 min (*p* < 0.025). None of the comparisons between groups across time were significantly different. A two‐way repeated‐measures ANOVA for GLU iAUC revealed a main effect of group (*F* = 4.605, *p* = 0.045), no effect of trial (*F* = 3.897, *p* = 0.0631), or trial*group interaction (*F* = 0.9827, *p* = 0.3340). GLU iAUC in the control trials tended to be lower compared to the exercise trials (mean difference: −40.89, 95% CI: −84.25 to 2.462). CU had lower iAUC values than NU (mean difference: −112.3, 95% CI: −221.8 to −2.765) (Figure 4d).

Total cholesterol (TC) was analyzed using a linear mixed model, which unveiled a main effect for time (*F* = 6.533, *p* = 0.0001), but no main effect of trial (*F* = 1.653, *p* = 0.2062) or main effect of group (*F* = 1.12, *p* = 0.3031) (Figure 5a). There was no significant time*trial (*F* = 1.214, *p* = 0.3117) or trial*group (*F* = 0.09, *p* = 0.7674) interaction, although the time*group interaction was significant (*F* = 3.12, *p* = 0.0197). The full interaction for time*trial*group was not significant (*F* = 1.292, *p* = 0.2806). Post hoc analysis of the time*group interaction revealed that NU had lower TC levels at Baseline compared to 30 min (*p* = 0.0032) and lower TC levels at 180 min compared to 120 min (*p* = 0.0037). None of the comparisons within CU and between CU and NU were significant. A two‐way repeated measures ANOVA for the TC iAUC data revealed a main effect of group (*F* = 4.594, *p* = 0.0452), but no main effect of trial (*F* = 1.019, *p* = 0.3253) or trial*group interaction (*F* = 0.148, *p* = 0.7048). CU had a lower TC iAUC compared to NU (mean difference:−58.13, 95% CI: −114.9 to −1.365). Pairwise comparisons did not reach statistical significance (Figure 5b).

Low‐density lipoprotein cholesterol (LDL) was analyzed using a linear mixed model, which unveiled a main effect for time (*F* = 8.056, *p* < 0.0001) but no main effect of trial (*F* = 0.7381, *p* = 0.3465) or group (*F* = 1.259, *p* = 0.2759) (Figure 5c). The time*trial (*F* = 2.046, *p* = 0.1249), time*group (*F* = 1.511, *p* = 0.2074), trial*group (*F* = 0.6524, *p* = 0.4292), and time*trial*group (*F* = 1.707, *p* = 0.1572) interactions were not statistically significant. Post hoc analyses revealed that Baseline LDL levels in the control and exercise trials were significantly lower than 30 and 60 min (*p* < 0.035 for all). Further, LDL levels at 30 and 60 min were significantly higher than 180 min in the exercise trial (*p* < 0.035 for both). A two‐way repeated‐measures ANOVA for the LDL iAUC data revealed no main effect of trial (*F*_1,19_ = 1.196, *p* = 0.2879), a significant main effect of group (*F*_1,19_ = 5.507, *p* = 0.0299), and no trial*group interaction (*F*_1,19_ = 0.4269, *p* = 0.5213). CU had a lower LDL iAUC compared to NU (mean difference: −27.19, 95% CI: −51.44 to −2.939). However, none of the pairwise comparisons reached statistical significance (Figure 5d).

High‐density lipoprotein cholesterol (HDL) was analyzed using a linear mixed model, which unveiled a main effect for time (*F* = 2.783, *p* = 0.0325), but no main effect of trial (*F* = 0.019, *p* = 0.8088) or main effect of group (*F* = 0.004, *p* = 0.9473) (Figure 5e). There was no significant time*trial (*F* = 1.759, *p* = 0.1752), time*group (*F* = 0.8377, *p* = 0.5055), or trial*group (*F* = 4.284, *p* = 0.0524) interaction. The full interaction for time*trial*group was significant (*F* = 3.182, *p* = 0.0179). None of the post hoc comparisons reached statistical significance. A two‐way repeated‐measures ANOVA for HDL iAUC data revealed no main effect of trial (*F*_1,19_ = 0.705, *p* = 0.4115), group (*F*_1,19_ = 0.6885, *p* = 0.4170), or trial*group interaction (*F*_1,19_ = 0.1587, *p* = 0.6948) (Figure 5f).

Mean arterial pressure (MAP) data was analyzed using a linear mixed model, which unveiled a main effect for time (*F* = 2.937, *p* = 0.0255) but no main effects for trial (*F* = 0.1564, *p* = 0.5048) or group (*F* = 0.3473, *p* = 0.5622) (Figure 6a). The time*trial (*F*_2.613,52.25_ = 0.3034, *p* = 0.7956), time*group (*F* = 0.8248, p = 0.5132), trial*group (*F* = 0.1772, *p* = 0.6783), and full time*trial*group (*F* = 1.015, *p* = 0.4049) interactions were not significant. Post hoc analysis revealed that NU had higher MAP values at 30 min compared to 180 min (*p* = 0.0089). No other comparisons were significant. A two‐way repeated‐measures ANOVA for MAP iAUC revealed no main effect of trial (*F* = 0.2496, *p* = 0.6228), group (*F* = 3.905, *p* = 0.0621), or trial*group interaction (*F* = 0.3712, *p* = 0.5492). CU did exhibit a higher MAP iAUC compared to NU (mean difference: +150.3, 95% CI: −8.35 to 309) (Figure 6b).

## 4 DISCUSSION

The purpose of this study was to compare the postprandial responses between cannabis users and nonusers using an MTT and determine if cannabis users responded differently when prior exercise is performed compared to nonusers. The primary findings from our study were as follows: (1) prior exercise restores fat oxidation in cannabis users, at the expense of metabolic flexibility; (2) prior exercise increases triglyceride metabolism to similar levels in cannabis users and nonusers; and (3) prior exercise does not impact MAP in cannabis users. These data suggest that cannabis users may be more sensitive to the metabolic effects of prior exercise, as opposed to the cardiovascular effects. These data do not support our hypotheses that cannabis users would have exaggerated PPL in resting conditions compared to nonusers, and that this response would not be improved with prior exercise.

Little data exist regarding the metabolic health of cannabis users, beyond epidemiological studies. Most of these studies report fasting data for various biomarkers in cannabis users compared to never‐ and past‐users (Bancks et al., 2015; Muniyappa et al., 2013; Penner et al., 2013; Vidot et al., 2016). Data from these studies report fasting levels of triglycerides between 1.0 and 1.42 mmol⋅L^−1^, which is higher than the mean fasting values observed in this study (0.6–0.77 mmol⋅L^−1^) (Bancks et al., 2015; Muniyappa et al., 2013; Penner et al., 2013; Vidot et al., 2016). Prior research also reveals glucose values between 5 and 5.5 mmol⋅L^−1^, which are also higher than the values observed in this study (4.47–4.65 mmol⋅L^−1^) (Bancks et al., 2015; Muniyappa et al., 2013; Penner et al., 2013; Vidot et al., 2016). Studies have also reported LDL cholesterol values between 2.2 and 2.8 mmol⋅L^−1^, with HDL values between 1.3 and 1.49, depending on sex (Bancks et al., 2015; Muniyappa et al., 2013; Penner et al., 2013; Vidot et al., 2016). LDL values in this study were similar to published values (2.73–2.77 mmol⋅L^−1^), as was HDL (1.38–1.44 mmol⋅L^−1^). Total cholesterol values in this study (4.56–4.58 mmol⋅L^−1^) were on the upper end of means reported in the literature (3.9–4.6 mmol⋅L^−1^) (Bancks et al., 2015; Muniyappa et al., 2013; Penner et al., 2013; Vidot et al., 2016). In sum, our data suggest that young, active cannabis users have normal glucose and triglyceride metabolism, but may experience alterations in cholesterol metabolism. Furthermore, epidemiological research has generally, though not always, reported lower BMI values in cannabis users (Clark et al., 2018; Sabia et al., 2017).

The mechanism(s) for the discrepancy between epidemiological data and acute studies are not clear. It has been speculated that cannabis users may have increased metabolic rates, but this data are purely speculative, and we observed no indication of this in this study (Clark et al., 2018). Another secondary analysis of a larger trial reported higher levels of palmitic, palmitoleic, and oleic acids in the serum of cannabis users compared to nonusers; in addition, the authors estimated de novo lipogenesis (DNL) from the ratio of palmitic acid to linoleic acid and reported higher DNL levels in cannabis users (Cisbani et al., 2022). The authors speculated that elevated serum glucose could have influenced palmitic acid production, though there was no difference between cannabis users and nonusers (Cisbani et al., 2022). Furthermore, pyruvate and lactate—the end products of glycolysis—were downregulated, which suggests the possibility that they were more rapidly converted into palmitic acid in cannabis users (Cisbani et al., 2022). However, this study did not control for habitual dietary intake or physical activity before blood collection nor were participants fasted for their blood draw (Cisbani et al., 2022). Finally, a rodent study may shed some light on the discrepancies in the literature. In that study, adolescent mice exposed to THC presented as apparently healthy adults, with lower levels of body fat, increased fat oxidation, resistance to diet‐induced obesity and dyslipidemia, and an improved lipid panel compared to non‐THC‐treated mice (Lin et al., 2023). However, these mice also exhibited altered thermoregulation, decreased cold and β‐receptor stimulated lipolysis, and an increased expression of skeletal muscle proteins and other abnormalities in adipose tissue (Lin et al., 2023). Future work should consider conducting skeletal muscle and adipose tissue biopsies during meal challenges to explore whether similar findings would be observed in humans.

Prior bouts of exercise are known to improve triglyceride metabolism after a high‐fat meal or MTT (Pearson et al., 2020; Rogers et al., 2023). In healthy adults, data indicates that non‐fasting triglycerides may be better predictors of CVD than fasting markers (Keirns et al., 2021). Exercise is well known to influence PPL in response to an MTT, with a recent meta‐analysis reporting exercise has a moderate effect (*d* = −0.47) on the total triglyceride response to a meal that was also moderate (*d* = −0.40) when expressed as an iAUC (Pearson et al., 2022). Prior exercise increases skeletal muscle lipoprotein‐lipase activity while simultaneously reducing secretion of VLDL from the liver (Kiens & Lithell, 1989; Pearson et al., 2022). To the authors' knowledge, MTT data in cannabis users is lacking. However, older and newer data illustrate that tobacco smokers have impaired post‐prandial lipid metabolism compared to nonsmokers (Alotaibi et al., 2021; Axelsen et al., 1995) in resting conditions. Using a similarly designed study, but utilizing two test meals, Alotaibi and colleagues recently demonstrated that even young tobacco smokers have altered responses to an MTT compared to nonsmokers (Alotaibi et al., 2021). It is interesting that postprandial fat oxidation was suppressed in the control condition in CU compared to the NU, as this has not been reported previously despite the similarities between cannabis and tobacco smoke (Graves et al., 2020). Exercise, however, appeared to counteract the suppression of lipolysis in CU—but at the expense of metabolic flexibility postprandially. This paradox provides an interesting area for future investigation and highlights the importance of future studies incorporating measurements of insulin and other metabolic hormones.

The lack of an impact of prior exercise on MAP in the cannabis users is interesting. As one of the primary components of cannabis, THC is a well‐known acute stimulator of the sympathetic nervous system. One of the most robust responses to acute cannabis use is increased heart rate (Cheung et al., 2022), but the effects of cannabis use on blood pressure are less clear. For example, despite an expected increase in peripheral vasoconstriction in response to cannabis exposure, some research has shown increased blood flow to the limbs (Jones, 2002). Recent work in cannabis users revealed that while there were no differences in cardiac morphology, systolic and diastolic function, and flow‐mediated dilation, cannabis users had higher pulse wave velocity values and reduced apical rotation compared to nonusers (Cheung et al., 2021). These authors expanded on their cross‐sectional results by measuring muscle sympathetic nerve activation (MSNA) in response to cannabis inhalation. It was reported that acute inhalation increased heart rate, MAP, SBP, DBP, cardiac output, and vascular conductance without impacting stroke volume and respiratory rate (Cheung et al., 2022). Simultaneously, MSNA was suppressed, and the authors reported these results were similar to cigarette consumption (Cheung et al., 2022). The conflict of the sympathetic and parasympathetic nervous systems in response to tobacco or cannabis smoking could potentially cause stress to the cardiovascular system that predisposes it to CVD, but this remains speculative.

## 5 STRENGTHS AND LIMITATIONS

Strengths of this study include the randomized crossover nature and participant control and standardization. Limitations to interpretation include lack of standardized meals the day before the MTT and no objective measure of physical activity the day before each MTT. Assessment of insulin, nonesterified fatty acids, C‐reactive protein, and other biomarkers would have allowed us to capture a better view of the participants' health during the MTT and also provide some mechanistic insight into our observations. Cannabis use was self‐reported, and we did not confirm this with a drug test. Cannabis users exercised at a significantly higher percentage of the VT compared to nonusers, which may be a limitation; however, heart rate, VO_2_, and blood lactate were not different, so it is safe to assume that the physiological cost of exercise was not different between the groups. Finally, this study assessed the responses to a single meal. Future research, including studies planned by the research team, should incorporate multiple meals or days of free‐living observation to capture a more holistic view of metabolic health in cannabis users. Given that users of tobacco and/or cannabis products use multiple times per day, research that factored this into the design of the study may shed light on how repeated exposure influences metabolic health.

## 6 CONCLUSIONS

This study examined the impact of cannabis use on PPL and the impact of exercise on PPL in this population. Our results indicate that young, otherwise healthy and active cannabis users have suppressed fat oxidation after an MTT in resting conditions, and that exercise restores fat oxidation in this population. Preliminary data suggest that triglyceride metabolism may also be improved to similar levels seen in NU. Future work should consider focusing on older, less active cannabis users to determine if they have impaired postprandial responses to meals that may increase their risks of cardiovascular and metabolic diseases.

## AUTHOR CONTRIBUTIONS

MMS conceived and designed the research, obtained funding, performed experiments, analyzed data, interpreted results of experiments, prepared figures, drafted the article, edited and revised the article, and approved the final version of the article. MP, GH, CA, and CM performed experiments and assisted with article preparation and editing. All authors approved the final version of the article.

## FUNDING INFORMATION

This research was funded by a California State University Program for Education & Research in Biotechnology (CSUPERB), New Investigator Award (to MMS).

## CONFLICT OF INTEREST STATEMENT

The authors declare no conflicts of interests.

## Data Availability

Data are available from the corresponding author upon reasonable request.

## References

[phy215968-bib-0001] Alotaibi, T. F. , Thackray, A. E. , Roberts, M. J. , Alanazi, T. M. , Bishop, N. C. , Wadley, A. J. , King, J. A. , O'Donnell, E. , Steiner, M. C. , Singh, S. J. , & Stensel, D. J. (2021). Acute running and coronary heart disease risk markers in male cigarette smokers and nonsmokers: A randomized crossover trial. Medicine and Science in Sports and Exercise, 53(5), 1021–1032. 33196606 10.1249/MSS.0000000000002560 PMC8048727

[phy215968-bib-0002] Auer, R. , Sidney, S. , Goff, D. , Vittinghoff, E. , Pletcher, M. J. , Allen, N. B. , Reis, J. P. , Lewis, C. E. , Carr, J. , & Rana, J. S. (2018). Lifetime marijuana use and subclinical atherosclerosis: The coronary artery risk development in Young adults (CARDIA) study. Addiction, 113(5), 845–856. 29168268 10.1111/add.14110

[phy215968-bib-0003] Axelsen, M. , Eliasson, B. , Joheim, E. , Lenner, R. A. , Taskinen, M. R. , & Smith, U. (1995). Lipid intolerance in smokers. Journal of Internal Medicine, 237(5), 449–455. 7738484 10.1111/j.1365-2796.1995.tb00869.x

[phy215968-bib-0004] Bancks, M. P. , Auer, R. , Carr, J. J. , Goff, D. C. , Kiefe, C. , Rana, J. S. , Reis, J. , Sidney, S. , Terry, J. G. , & Schreiner, P. J. (2018). Self‐reported marijuana use over 25 years and abdominal adiposity: The coronary artery risk development in Young adults (CARDIA) study. Addiction, 113(4), 689–698. 29127726 10.1111/add.14097 PMC5847434

[phy215968-bib-0005] Bancks, M. P. , Pletcher, M. J. , Kertesz, S. G. , Sidney, S. , Rana, J. S. , & Schreiner, P. J. (2015). Marijuana use and risk of prediabetes and diabetes by middle adulthood: The coronary artery risk development in Young adults (CARDIA) study. Diabetologia, 58(12), 2736–2744. 26364621 10.1007/s00125-015-3740-3 PMC4631659

[phy215968-bib-0006] Beaver, W. L. , Wasserman, K. , & Whipp, B. J. (1986). A new method for detecting anaerobic threshold by gas exchange. Journal of Applied Physiology (Bethesda, MD: 1985), 60(6), 2020–2027. 3087938 10.1152/jappl.1986.60.6.2020

[phy215968-bib-0007] Benowitz, N. L. , & Jones, R. T. (1975). Cardiovascular effects of prolonged delta‐9‐tetrahydrocannabinol ingestion. Clinical Pharmacology and Therapeutics, 18(3), 287–297. 1164818 10.1002/cpt1975183287

[phy215968-bib-0008] Blaak, E. E. , Antoine, J. M. , Benton, D. , Björck, I. , Bozzetto, L. , Brouns, F. , Diamant, M. , Dye, L. , Hulshof, T. , Holst, J. J. , Lamport, D. J. , Laville, M. , Lawton, C. L. , Meheust, A. , Nilson, A. , Normand, S. , Rivellese, A. A. , Theis, S. , Torekov, S. S. , & Vinoy, S. (2012). Impact of postprandial glycaemia on health and prevention of disease. Obesity Reviews, 13(10), 923–984. 22780564 10.1111/j.1467-789X.2012.01011.x PMC3494382

[phy215968-bib-0009] Borg, G. A. (1982). Psychophysical bases of perceived exertion. Medicine and Science in Sports and Exercise, 14(5), 377–381. 7154893

[phy215968-bib-0010] Cheung, C. P. , Coates, A. M. , Millar, P. J. , & Burr, J. F. (2021). Habitual cannabis use is associated with altered cardiac mechanics and arterial stiffness, but not endothelial function in young healthy smokers. Journal of Applied Physiology, 130(3), 660–670. 33444123 10.1152/japplphysiol.00840.2020

[phy215968-bib-0011] Cheung, C. P. , Nardone, M. , Lee, J. B. , Baker, R. E. , Millar, P. J. , & Burr, J. F. (2022). Cannabis inhalation acutely reduces muscle sympathetic nerve activity in humans. Circulation, 146(25), 1972–1974. 36314198 10.1161/CIRCULATIONAHA.122.062564

[phy215968-bib-0012] Cisbani, G. , Koppel, A. , Metherel, A. H. , Smith, M. E. , Aji, K. N. , Andreazza, A. C. , Mizrahi, R. , & Bazinet, R. P. (2022). Serum lipid analysis and isotopic enrichment is suggestive of greater lipogenesis in young long‐term cannabis users: A secondary analysis of a case–control study. Lipids, 57(2), 125–140. 35075659 10.1002/lipd.12336 PMC8923992

[phy215968-bib-0013] Clark, T. M. , Jones, J. M. , Hall, A. G. , Tabner, S. A. , & Kmiec, R. L. (2018). Theoretical explanation for reduced body mass index and obesity rates in *cannabis* users. Cannabis and Cannabinoid Research, 3(1), 259–271. 30671538 10.1089/can.2018.0045 PMC6340377

[phy215968-bib-0014] Frayn, K. N. (1983). Calculation of substrate oxidation rates in vivo from gaseous exchange. Journal of Applied Physiology, 55(2), 628–634. 6618956 10.1152/jappl.1983.55.2.628

[phy215968-bib-0015] Gilbertson, N. M. , Eichner, N. Z. M. , Francois, M. , Gaitán, J. M. , Heiston, E. M. , Weltman, A. , & Malin, S. K. (2018). Glucose tolerance is linked to postprandial fuel use independent of exercise dose. Medicine and Science in Sports and Exercise, 50(10), 2058–2066. 29762253 10.1249/MSS.0000000000001667

[phy215968-bib-0016] Goodpaster, B. H. , & Sparks, L. M. (2017). Metabolic flexibility in health and disease. Cell Metabolism, 25(5), 1027–1036. 28467922 10.1016/j.cmet.2017.04.015 PMC5513193

[phy215968-bib-0017] Graves, B. M. , Johnson, T. J. , Nishida, R. T. , Dias, R. P. , Savareear, B. , Harynuk, J. J. , Kazemimanesh, M. , Olfert, J. S. , & Boies, A. M. (2020). Comprehensive characterization of mainstream marijuana and tobacco smoke. Scientific Reports, 10(1), 7160. 32345986 10.1038/s41598-020-63120-6 PMC7188852

[phy215968-bib-0018] Hardy, C. J. , & Rejeski, W. J. (1989). Not what, but how one feels: The measurement of affect during exercise. Journal of Sport & Exercise Psychology, 11(3), 304–317.

[phy215968-bib-0019] Hollister, L. E. , & Reaven, G. M. (1974). Delta‐9‐tetrahydrocannabinol and glucose tolerance. Clinical Pharmacology and Therapeutics, 16(2), 297–302. 4851289 10.1002/cpt1974162297

[phy215968-bib-0020] Hurren, N. M. , Eves, F. F. , & Blannin, A. K. (2011). Is the effect of prior exercise on postprandial lipaemia the same for a moderate‐fat meal as it is for a high‐fat meal? The British Journal of Nutrition, 105(4), 506–516. 21073762 10.1017/S0007114510003995

[phy215968-bib-0021] Jakob, J. , Von Wyl, R. , Stalder, O. , Pletcher, M. J. , Vittinghoff, E. , Tal, K. , Rana, J. S. , Sidney, S. , Reis, J. P. , & Auer, R. (2021). Cumulative marijuana use and carotid intima‐media thickness at middle age: The CARDIA study. The American Journal of Medicine, 134(6), 777–787.e9. 33359272 10.1016/j.amjmed.2020.11.026

[phy215968-bib-0022] Jeukendrup, A. E. , & Wallis, G. A. (2005). Measurement of substrate oxidation during exercise by means of gas exchange measurements. International Journal of Sports Medicine, 26(Suppl 1), S28–S37. 15702454 10.1055/s-2004-830512

[phy215968-bib-0023] Jones, R. T. (2002). Cardiovascular system effects of marijuana. Journal of Clinical Pharmacology, 42(S1), 58S–63S. 12412837 10.1002/j.1552-4604.2002.tb06004.x

[phy215968-bib-0024] Keirns, B. H. , Sciarrillo, C. M. , Koemel, N. A. , & Emerson, S. R. (2021). Fasting, non‐fasting and postprandial triglycerides for screening cardiometabolic risk. Journal of Nutritional Science, 10, e75. 34589207 10.1017/jns.2021.73 PMC8453457

[phy215968-bib-0025] Kendzierski, D. , & DeCarlo, K. J. (1991). Physical activity enjoyment scale: Two validation studies. Journal of Sport & Exercise Psychology, 13(1), 50–64.

[phy215968-bib-0026] Kiens, B. , & Lithell, H. (1989). Lipoprotein metabolism influenced by training‐induced changes in human skeletal muscle. The Journal of Clinical Investigation, 83(2), 558–564. 2643634 10.1172/JCI113918 PMC303715

[phy215968-bib-0027] Kolovou, G. D. , Mikhailidis, D. P. , Kovar, J. , Lairon, D. , Nordestgaard, B. G. , Ooi, T. C. , Perez‐Martinez, P. , Bilianou, H. , Anagnostopoulou, K. , & Panotopoulos, G. (2011). Assessment and clinical relevance of non‐fasting and postprandial triglycerides: An expert panel statement. Current Vascular Pharmacology, 9(3), 258–270. 21314632 10.2174/157016111795495549

[phy215968-bib-0028] Lin, L. , Jung, K. M. , Lee, H. L. , Le, J. , Colleluori, G. , Wood, C. , Palese, F. , Squire, E. , Ramirez, J. , Su, S. , Torrens, A. , Fotio, Y. , Tang, L. , Yu, C. , Yang, Q. , Huang, L. , DiPatrizio, N. , Jang, C. , Cinti, S. , & Piomelli, D. (2023). Adolescent exposure to low‐dose THC disrupts energy balance and adipose organ homeostasis in adulthood. Cell Metabolism, 35(7), 1227–1241.e7. 37267956 10.1016/j.cmet.2023.05.002 PMC10524841

[phy215968-bib-0029] Meier, M. H. , Pardini, D. , Beardslee, J. , & Matthews, K. A. (2019). Associations between cannabis use and Cardiometabolic risk factors: A longitudinal study of men. Psychosomatic Medicine, 81(3), 281–288. 30589665 10.1097/PSY.0000000000000665 PMC6443484

[phy215968-bib-0030] Muniyappa, R. , Sable, S. , Ouwerkerk, R. , Mari, A. , Gharib, A. M. , Walter, M. , Courville, A. , Hall, G. , Chen, K. Y. , Volkow, N. D. , Kunos, G. , Huestis, M. A. , & Skarulis, M. C. (2013). Metabolic effects of chronic cannabis smoking. Diabetes Care, 36(8), 2415–2422. 23530011 10.2337/dc12-2303 PMC3714514

[phy215968-bib-0031] Narang, B. J. , Atkinson, G. , Gonzalez, J. T. , & Betts, J. A. (2020). A tool to explore discrete‐time data: The time series response analyser. International Journal of Sport Nutrition and Exercise Metabolism, 30(5), 374–381. 32726749 10.1123/ijsnem.2020-0150

[phy215968-bib-0032] Ong, L. Q. , Bellettiere, J. , Alvarado, C. , Chavez, P. , & Berardi, V. (2021). Cannabis use, sedentary behavior, and physical activity in a nationally representative sample of US adults. Harm Reduction Journal, 18(1), 48. 33926458 10.1186/s12954-021-00496-2 PMC8086340

[phy215968-bib-0033] Page, R. L. , Allen, L. A. , Kloner, R. A. , Carriker, C. R. , Martel, C. , Morris, A. A. , Piano, M. R. , Rana, J. S. , Saucedo, J. F. , American Heart Association Clinical Pharmacology Committee and Heart Failure and Transplantation Committee of the Council on Clinical Cardiology , Council on Cardiovascular and Stroke Nursing , Council on Cardiovascular and Stroke Nursing , Council on Epidemiology and Prevention , Council on Lifestyle and Cardiometabolic Health , & Council on Quality of Care and Outcomes Research . (2020). Medical marijuana, recreational cannabis, and cardiovascular health: A scientific Statement from the American Heart Association. Circulation, 142(10), e131–e152. 10.1161/CIR.0000000000000883 32752884

[phy215968-bib-0034] Pearson, R. C. , Cogan, B. , Garcia, S. A. , & Jenkins, N. T. (2022). Effect of prior exercise on postprandial Lipemia: An updated meta‐analysis and systematic review. International Journal of Sport Nutrition and Exercise Metabolism, 32(6), 501–518. 36028221 10.1123/ijsnem.2022-0043

[phy215968-bib-0035] Pearson, R. C. , Olenick, A. A. , Green, E. S. , & Jenkins, N. T. (2020). Tabata‐style functional exercise increases resting and postprandial fat oxidation but does not reduce triglyceride concentrations. Experimental Physiology, 105(3), 468–476. 31916294 10.1113/EP088330

[phy215968-bib-0036] Penner, E. A. , Buettner, H. , & Mittleman, M. A. (2013). The impact of marijuana use on glucose, insulin, and insulin resistance among US adults. The American Journal of Medicine, 126(7), 583–589. 23684393 10.1016/j.amjmed.2013.03.002

[phy215968-bib-0037] Podolsky, S. , Pattavina, C. G. , & Amaral, M. A. (1971). Effect of marijuana on the glucose‐tolerance test*. Annals of the New York Academy of Sciences, 191(1), 54–60.

[phy215968-bib-0038] Ponce Orellana, C. , Roldan, P. , Sibbitt, W. , Qualls, C. , & Roldan, C. (2015). Abstract 577: Medical marijuana use: Effect on lipid metabolism and atherosclerosis. Arteriosclerosis, Thrombosis, and Vascular Biology, 35(suppl_1), A577.

[phy215968-bib-0039] Reis, J. P. , Auer, R. , Bancks, M. P. , Goff, D. C. , Lewis, C. E. , Pletcher, M. J. , Rana, J. S. , Shikany, J. M. , & Sidney, S. (2017). Cumulative lifetime marijuana use and incident cardiovascular disease in middle age: The coronary artery risk development in Young adults (CARDIA) study. American Journal of Public Health, 107(4), 601–606. 28207342 10.2105/AJPH.2017.303654 PMC5343712

[phy215968-bib-0040] Robergs, R. A. , Dwyer, D. , & Astorino, T. (2010). Recommendations for improved data processing from expired gas analysis indirect calorimetry. Sports medicine (Auckland, N.Z.), 40(2), 95–111. 20092364 10.2165/11319670-000000000-00000

[phy215968-bib-0041] Rogers, E. M. , Banks, N. F. , & Jenkins, N. D. M. (2023). Acute effects of daily step count on postprandial metabolism and resting fat oxidation: A randomized controlled trial. Journal of Applied Physiology (Bethesda, MD: 1985), 135(4), 812–822. 37560764 10.1152/japplphysiol.00052.2023

[phy215968-bib-0042] Ryan, M. F. , Grada, C. O. , Morris, C. , Segurado, R. , Walsh, M. C. , Gibney, E. R. , Brennan, L. , Roche, H. M. , & Gibney, M. J. (2013). Within‐person variation in the postprandial lipemic response of healthy adults. The American Journal of Clinical Nutrition, 97(2), 261–267. 23283501 10.3945/ajcn.112.047936

[phy215968-bib-0043] Sabia, J. J. , Swigert, J. , & Young, T. (2017). The effect of medical marijuana Laws on Body weight: Medical marijuana Laws and Body weight. Health Economics, 26(1), 6–34. 10.1002/hec.3267 26602324

[phy215968-bib-0044] Smith, L. , Sherratt, F. , Barnett, Y. , Cao, C. , Tully, M. A. , Koyanagi, A. , Jacob, L. , Soysal, P. , López Sánchez, G. F. , Shin, J. I. , & Yang, L. (2021). Physical activity, sedentary behaviour and cannabis use in 15,822 US adults: Cross‐sectional analyses from NHANES. Public Health, 193, 76–82. 33743217 10.1016/j.puhe.2021.01.018

[phy215968-bib-0045] Thompson, D. , Karpe, F. , Lafontan, M. , & Frayn, K. (2012). Physical activity and exercise in the regulation of human adipose tissue physiology. Physiological Reviews, 92(1), 157–191. 22298655 10.1152/physrev.00012.2011

[phy215968-bib-0046] Vidot, D. C. , Prado, G. , Hlaing, W. M. , Florez, H. J. , Arheart, K. L. , & Messiah, S. E. (2016). Metabolic syndrome among marijuana users in the United States: An analysis of National Health and nutrition examination survey data. The American Journal of Medicine, 129(2), 173–179. 26548604 10.1016/j.amjmed.2015.10.019 PMC4718895

[phy215968-bib-0047] Weiss, J. L. , Watanabe, A. M. , Lemberger, L. , Tamarkin, N. R. , & Cardon, P. V. (1972). Cardiovascular effects of delta‐9‐tetrahydrocannabinol in man. Clinical Pharmacology and Therapeutics, 13(5part1), 671–684. 4559810 10.1002/cpt1972135part1671

[phy215968-bib-0048] YorkWilliams, S. L. , Gust, C. J. , Mueller, R. , Bidwell, L. C. , Hutchison, K. E. , Gillman, A. S. , & Bryan, A. D. (2019). The new Runner's high? Examining relationships between cannabis use and exercise behavior in states with legalized cannabis. Frontiers in Public Health, 30(7), 99. 10.3389/fpubh.2019.00099 PMC6503143 31114776

[phy215968-bib-0049] Yu, E. A. , Le, N. A. , & Stein, A. D. (2021). Measuring postprandial metabolic flexibility to assess metabolic health and disease. The Journal of Nutrition, 151(11), 3284–3291. 34293154 10.1093/jn/nxab263 PMC8562077

